# An Introduction to the Adherence Promotion With Person-Centered Technology Project

**DOI:** 10.1093/geroni/igaa057.2263

**Published:** 2020-12-16

**Authors:** Michael Dieciuc, Dawn Carr, Zhe He, Shayok Chakraborty, Neil Charness, Mia Lustria, Ankita Singh, Walter Boot

**Affiliations:** Florida State University, Tallahassee, Florida, United States

## Abstract

The massive potential of cognitive training and longitudinal cognitive assessment to detect and prevent age-related cognitive decline and dementia will not be realized unless individuals are willing and able to engage with these protocols for an extended period of time. Unfortunately, similar to other health behaviors, adherence to home-based assessment and training is frequently poor. Addressing the gap between potential and realized benefits is an urgent goal as the population ages. APPT investigates these and related issues within samples of older adults with and without cognitive impairment. Ultimately, two randomized controlled trials will test whether an adaptive, tailored, and integrated technology-based adherence support system can boost adherence, with the ultimate goal being the early detection and treatment of age-related cognitive decline and dementia. Initial algorithm development and application to existing datasets will be presented that will inform the design of a smart reminder system that will later be assessed.

